# The Surgical Treatment of Varicose Veins

**Published:** 1908-09

**Authors:** Charles Greene Cumston

**Affiliations:** Boston, Mass.


### BUFFALO MEDICAL JOURNAL.

Vol. Lxiv. SEPTEMBER, 1908. No. 2

### ORIGINAL COMMUNICATIONS.

### The Surgical Treatment of Varicose Veins.

By CHARLES GREENE CUMSTON, M. D., Boston, Mass.

THE physician is very frequently consulted relative to a surgical operation for varicose veins and he frequently is at loss as to what advice he may properly give. The case may merely be one of capillary varices seated on the calves or on the thighs. This condition gives rise to itching, edema and cutaneous infections, and although quite uncomfortable, surgical treatment is not to be resorted to. Their dissemination, small size and site, having no well defined territory, is quite in opposition to an operative interference.

In cases of varix of large veins the question is quite different. One should distinguish between patients presenting this condition with a normal vascular tension and those having a high tension. In varices with a high tension, the blood pressure, which is variable according to whether the subject is standing or lying down, quiet or exerting violent muscular efforts, varies in the central end of the saphenous between two to twenty centimeters of mercury. In certain strong subjects the tension may be even greater than twenty-five centimeters.

This enormous exaggeration of the venous tension can only be produced on the condition that the twelve or fifteen valves seated inside the vein are forced and that the weight of the blood column falls directly back from the iliac veins and the vena cava toward the small venous trunks of the foot. Trendelenburg has shown how varices with high tension can be recognised. The patient being seated or lying down, the diseased leg is raised and the blood being pushed out of the varices gives rise to a complete collapse of the dilated vessels. If now one compresses the trunk of the saphenous with the finger and the patient is made to stand, the veins of the leg will not fill up, while the blood will rush from above as soon as the pressure is removed. In these cases resection of the vein will be useful, more frequently resection of the trunk of the internal saphenous, more infrequently that of a mass of dilated veins. The latter operation should be resorted to if this large mass becomes painful and increases in size.

The operation is not serious and can be done with local anesthesia. One should also resect any ampullar dilatation of the internal saphenous at its entrance into the fascia, a condition which has sometimes been mistaken for a femoral hernia. Simple ligature may sometimes be sufficient, but as it is not any more serious by far the surest method is to resect the vein between two ligatures. All this does not mean that varices of low tension are inoffensive, because phlebitis, disturbances in walking and edema may appear and recur with the greatest obstinacy; the repetition of these accidents will occasionally oblige one to operate, but generally speaking this is exceptional.

In spite of every effort recurrences may take place. Complications make their appearance, such as central or peripheral thrombosis and early or late embolus. The possibility of these complications have caused certain surgeons to abstain from operating, because they believe that an operation in no way changes the intimate causes of the phlebectasia and that the suppression of the superficial venous currents can only result in forcing the blood of the limb into the deep veins and thus increase their dilatation. It has also been upheld that when phlebitis appears spontaneously it is in itself a curative process. This, however, is not always the case, because the phlebitis is not necessarily of the obliterating type; consequently the blood current can become reëstablished and the patient still remains exposed to all the possible complications of a future attack. On the other hand if a patient operated upon is menaced with thrombosis or embolus, the one not operated upon is not immune from this possibility. And, lastly, the functional troubles are certainly more especially due to varices of the superficial veins than of the deep ones, on which the muscular contraction exercises such a favorable action. According to Chretien the indications for operation should not be limited to pain and hemorrhage. If, in the well-to-do class, rest in the horizontal position is possible the above two mentioned complications may perhaps be the only absolute indication for operating, but in the poor, who are obliged to earn their livelihood by daily effort, the question is entirely different. The edema, ulcers, trophic disturbances and difficulty in walking are all conditions which justify or necessitate surgical interference. In the army the same conditions are found. The signs of a valvular insufficiency, a localised phlebitic thrombosis, ampullar dilatations which greatly impede the patient on account of their seat and size, attacks of neuralgia due to compression of the nerve filaments, are all conditions which render the case fit for surgical interference.

An ulcer may create contraindications for operating in certain cases, when this process is of long standing, accompanied by trophic disturbances in the skin and bone and has brought about atropic sclerosis in the structures of the limb. Under these circumstances any operative interference will result in a complete failure, because the ulcer will promptly recur on a badly nourished limb, whose nerves are choked by a hyperproduction of connective tissue and infection becomes more menacing after an operation has been done. If, on the other hand, the ulcer is recent, if the varices have acquired a high tension within a short time, if the limb does not present any trophic disturbances, one will be in the right to interfere surgically. The operation, however, will be a delicate one, because an ulcer is a nest for bacteria and it is difficult to render the lesion sufficiently clean so that one can operate in its neighborhood with safety. For this reason it is better to wait and when the ulcer is of recent date cicatrisation may be obtained after a more or less long lapse of time by proper hygienic precautions and dressings. Medical treatment will result in the cure of the ulcer and if later the varix continues to be a hindrance, it will then be time enough to treat it by resection.

871 BEACON STREET.

